# The Gynæcological Society

**Published:** 1900-05-05

**Authors:** 


### THE GYNÆCOLOGICAL SOCIETY.

In relation to the above remarks by Dr. Duncan it is worth while to refer to the proceedings of the last meeting of the Gynæcological Society, at which, in the course of a discussion upon a case of supra-vaginal hysterectomy for uterine myoma, the specimen from which was shown by Dr. Macnaughton-Jones, Dr. Frederick Edge remarked that, in view of the fact that some of these supposed myomata turned out to be sarcomatous in nature, he did not think it was justifiable to leave the cervix, since no one could tell beforehand whether the particular case would prove in the end to be sarcomatous or not. According then to Dr. Edge the operation of pan-hysterectomy should be performed in such cases. Commenting upon this in turn, Dr. Duncan disagreed with Dr. Edge, holding that in the vast majority of cases they had to do with a true myoma and not a sarcoma. Dr. Herbert Snow also took exception to the view laid down by Dr. Edge, maintaining that malignant degeneration of myomata was quite exceptional, and certainly did not occur in more than 5 per cent. of the cases. He did not think that the small risk of malignancy made it worth while to incur the increased danger of injury to the ureters which was involved in pan-hysterectomy. The president (Dr. Smyly), however, seemed to be in favour of pan-hysterectomy, although he confessed that if Dr. Duncan could show a mortality of only three in 68 cases he might well adhere to the plan by aid of which he had obtained such results.

